# Incidental Finding of Transthyretin Cardiac Amyloidosis During Coronary Artery Bypass Grafting

**DOI:** 10.7759/cureus.59466

**Published:** 2024-05-01

**Authors:** Narain Badhey, Antonia Nevias-Ida, Hemanth Badhey

**Affiliations:** 1 Cardiology, Touro College of Osteopathic Medicine, New York, USA; 2 Emergency Medicine, Touro College of Osteopathic Medicine, New York, USA; 3 Cardiology, St. Francis Hospital and Heart Center, Roslyn, USA

**Keywords:** cad: coronary artery disease, 3rd degree heart block, complex percutaneous coronary intervention, multivessel coronary artery disease (mvcad), single photon emission computed tomography (spect), technetium-99m pyrophosphate scan, infiltrative cardiomyopathy, cardio vascular disease, coronary artery bypass grafting(cabg), transthyretin amyloidosis

## Abstract

Transthyretin cardiac amyloidosis (ATTR-CA) is a condition characterized by extracellular deposition of misfolded transthyretin proteins in the myocardium and has been historically difficult to diagnose due to diverse clinical manifestations and nonspecific, variable electrocardiogram (ECG) and echocardiogram findings. Advancements in noninvasive cardiac imaging have led to significant increases in diagnoses of ATTR-CA. Once thought to be a rare condition, there is growing evidence to suggest that ATTR-CA is more prevalent than previously understood, prompting the need for early diagnosis and intervention. We outline the case of a 78-year-old male who presented to the emergency department with chest discomfort, shortness of breath, dizziness, and diaphoresis. He was found to have severe coronary artery disease (CAD) and intermittent complete heart block. Cardiac dysfunction was unable to be resolved by percutaneous coronary intervention (PCI) and thus the patient was referred for coronary artery bypass grafting (CABG). Intraoperatively, the patient's heart was found to be abnormally thickened and fibrosed. Biopsy of the cardiac tissue and evaluation using technetium-99m pyrophosphate scintigraphy, single-photon emission computed tomography, and liquid chromatography-tandem mass spectrometry revealed ATTR-CA. There is a need for fast and low-cost screening tools to allow for early identification of the disease. Diagnostic clues for cardiac amyloidosis include the presence of carpal tunnel syndrome, lumbar spinal stenosis, atrial fibrillation, treatment-resistant heart failure with preserved ejection fraction, and a thickened left ventricular wall. Given the presence of these red flag symptoms, clinicians should have a heightened index of suspicion for ATTR cardiac amyloidosis in elderly patients even when presenting in acute settings.

## Introduction

This case was presented as a poster presentation at the American College of Osteopathic Internists (ACOI) Annual Meeting in Tampa, Florida on October 13, 2023.

We present a case of wild-type transthyretin cardiac amyloidosis incidentally discovered during two-vessel coronary artery bypass grafting (CABG) conducted for the resolution of diastolic congestive heart failure (CHF). Transthyretin amyloidosis (ATTR) is a condition characterized by the extracellular deposition of misfolded transthyretin proteins in the myocardium, leading to morphological and functional changes in the heart [1]. Deposition of these proteins forms insoluble fibers that lead to thickening and stiffening of cardiac tissue [2]. Subsequent cardiac remodeling often leads to reduced filling of ventricles leading to diastolic dysfunction and restrictive cardiomyopathy. [3]

ATTR is subtyped based on the presence or absence of genetic mutations contributing to transthyretin misfolding. The hereditary variant of transthyretin cardiac amyloidosis (ATTRv-CA) is an autosomal dominant disease caused by a genetic mutation leading to the destabilization of transthyretin proteins [4]. Wild-type ATTR-CA (ATTRwt-CA), also known as age-related or senile cardiac amyloidosis, is characterized by misfolding of the theoretically normal wild-type transthyretin protein leading to amyloid deposition. The mechanism by which this misfolding occurs is not well elucidated but may involve post-translational modifications of transthyretin or inappropriate chaperone protein synthesis in the liver, resulting in failed clearance of misfolded transthyretin [5]. 

ATTR-CA has been historically difficult to diagnose due to diverse clinical manifestations and nonspecific, variable electrocardiogram (ECG) and echocardiogram findings [6]. Advancements in noninvasive cardiac imaging have led to significant increases in diagnoses of ATTR-CA [7]. Once thought to be a rare condition, recent research suggests that ATTR-CA may be significantly underdiagnosed and far more prevalent in the general population than previously assumed [8,9].

## Case presentation

**Figure 1:**
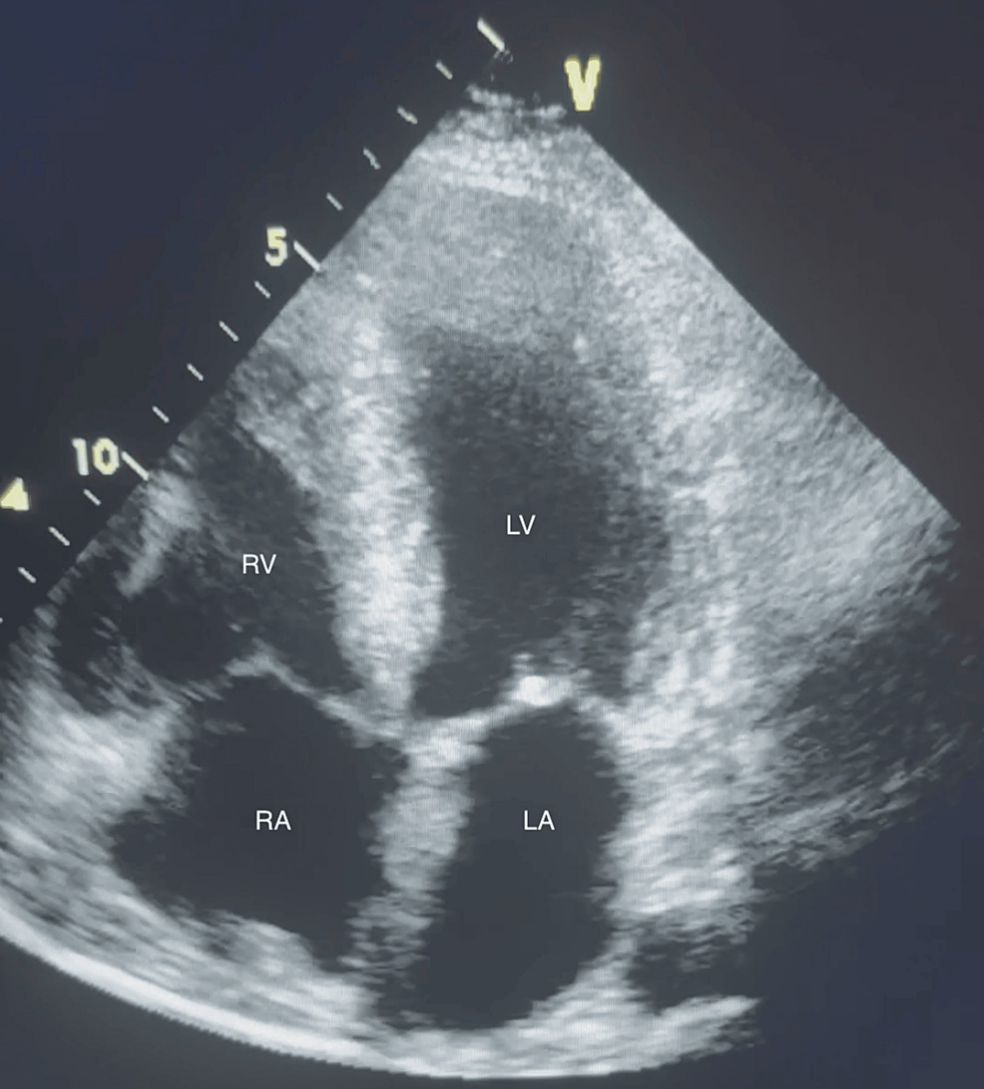
Apical four-chamber view on TTE 11 days prior to presentation, revealing biatrial enlargement LA: left atrium; LV: left ventricle; RA: right atrium; RV: right ventricle; TTE: transthoracic echocardiogram

**Figure 2:**
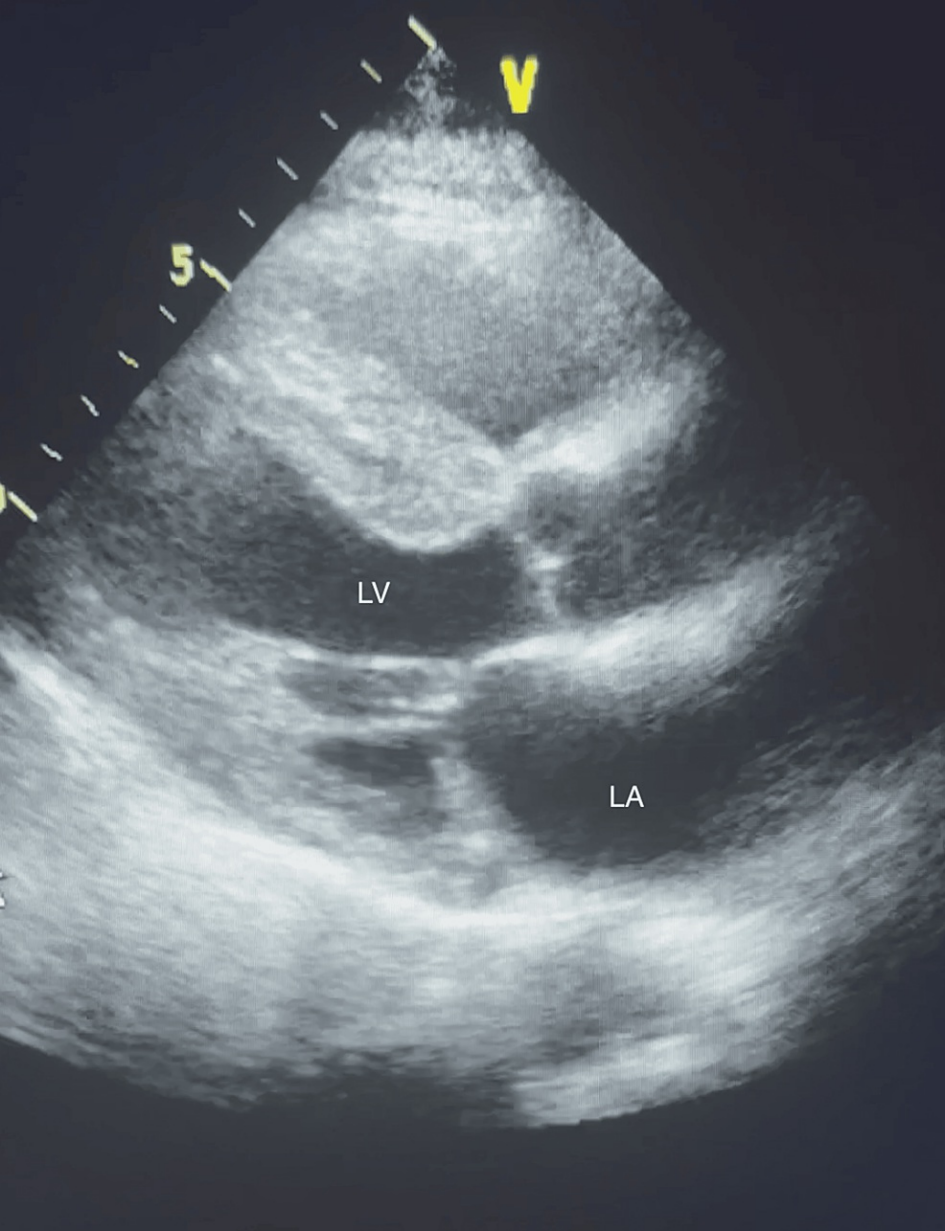
Parasternal long-axis view on TTE 11 days prior to presentation revealing left ventricular hypertrophy LA: left atrium; LV: left ventricle; TTE: transthoracic echocardiogram

A 78-year-old male presented to the emergency department with a 30-minute history of chest discomfort, shortness of breath, dizziness, and diaphoresis. The patient’s past medical history was significant for hypertension, hypercholesterolemia, insulin-dependent type II diabetes mellitus, elevated body mass index, lumbar spinal stenosis, and carpal tunnel syndrome with associated surgical release. The patient had no prior history of acute cardiac events but had several years of abnormal ECGs and was routinely evaluated and monitored by a cardiologist. The patient underwent transthoracic echocardiogram (TTE) testing 11 days prior to his presentation, revealing demonstrated biatrial enlargement (Figure 1), moderate mitral regurgitation, and concentric left ventricular hypertrophy (Figure 2) with preserved left ventricular systolic function.

The patient underwent evaluation in the emergency department where serial vital signs were measured, pertinent labs were drawn, ECG testing was conducted and a chest X-ray was performed. During the initial assessment, the patient was lightheaded and diaphoretic despite stable vitals and a SpO2 of 99% on room air. The patient was placed on bilevel positive airway pressure (BiPAP) and laboratory results revealed serum troponin I within normal limits of 0.020 ng/ml but an elevated serum pro-brain natriuretic peptide (pro-BNP) level of 843 pg/ml.

**Figure 3:**
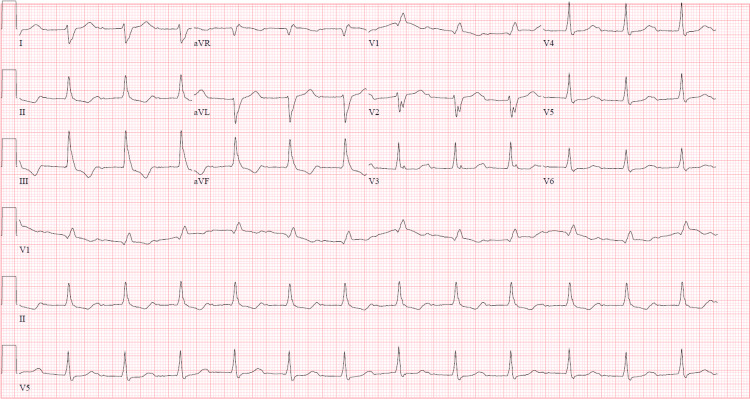
Initial ECG performed in ED demonstrating first degree-AV block, prolonged PR interval, right axis deviation, RBBB, ST-T wave changes in leads II, III, and aVF, and prolonged QT interval ECG: electrocardiogram; ED: emergency department; AV: atrioventricular; RBBB: right bundle branch block

**Figure 4:**
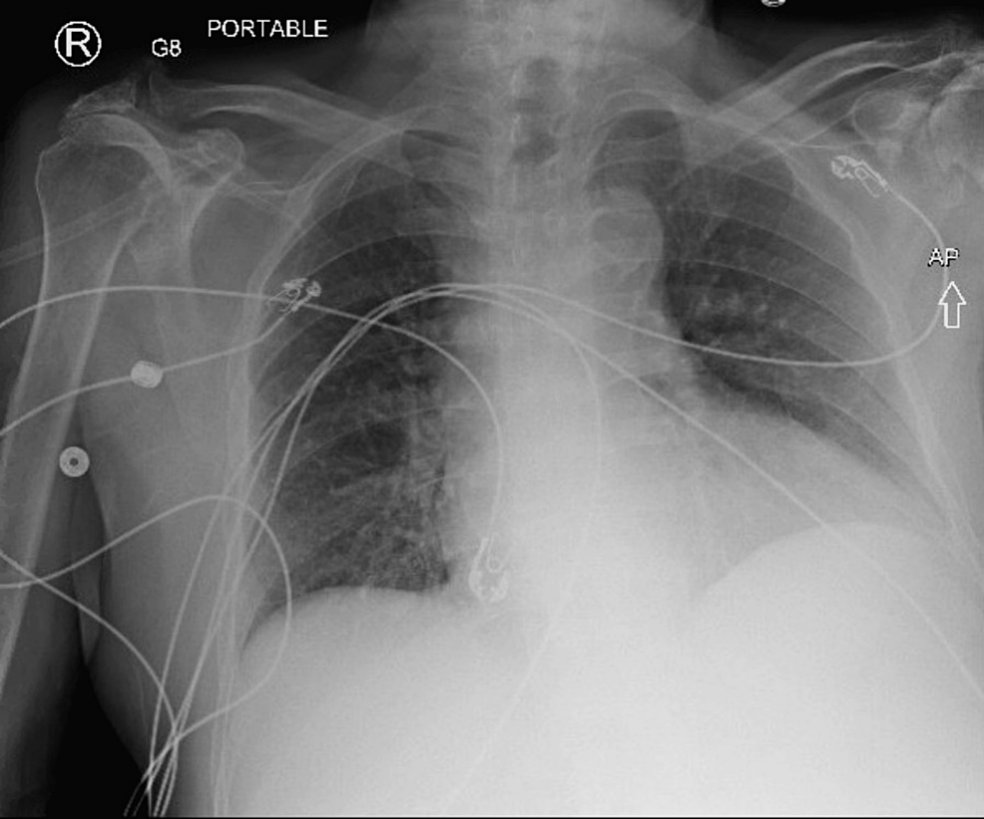
AP portable chest X-ray showing enlarged heart and low lung volumes AP: Anteroposterior

Initial ECG (Figure 3) was abnormal and the chest X-ray (Figure 4) revealed cardiomegaly with no active infiltrates.

**Figure 5:**
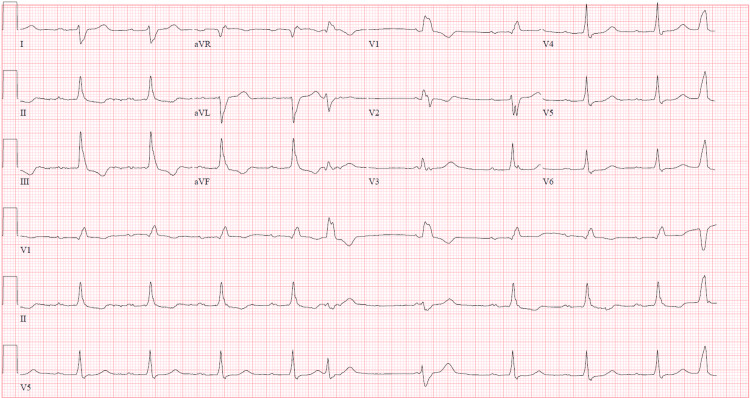
Serial ECG 2/2 demonstrating first-degree AV block, prolonged PR interval, right axis deviation, RBBB, ST-T wave changes in leads II, III, and aVF, prolonged QT interval, occasional APC with aberrancy, occasional PVC AV: atrioventricular; RBBB: right bundle branch block; APC: atrial premature complex; PVC: ventricular premature complex

Given the patient's abnormal ECG and cardiac symptoms, the patient was monitored in the ED, and serial ECGs (Figure 5) were conducted. The patient was then transferred from the ED to inpatient service, where he was weaned off BiPAP and given a dose of lorazepam. At this time the patient noted resolution of all symptoms and was being considered for discharge. After undergoing a cardiology assessment, the patient was recommended for cardiac catheterization.

**Figure 6:**
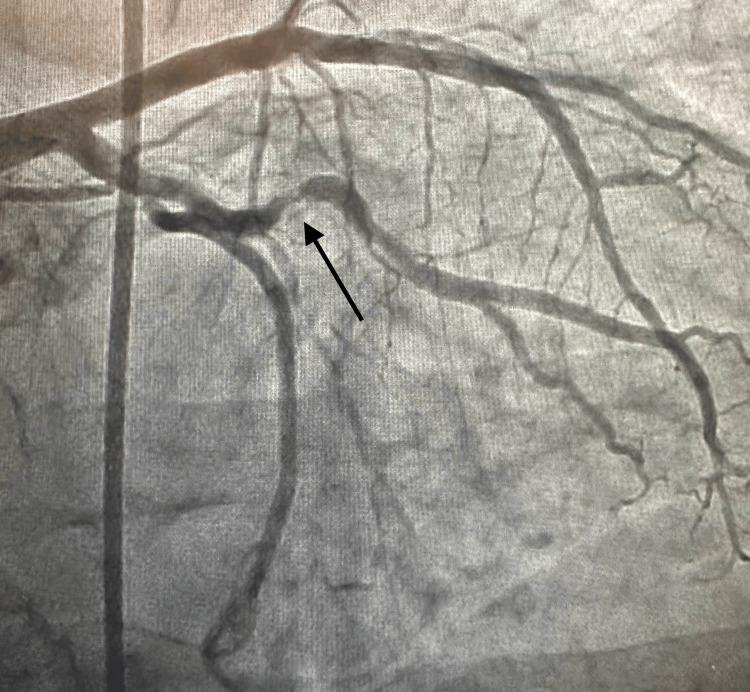
Coronary angiography revealing an eccentric lesion in the left circumflex artery The arrow denotes the area of stenosis in the mid-left circumflex artery. The region shows an eccentric lesion with 90% stenosis of the vessel.

**Figure 7:**
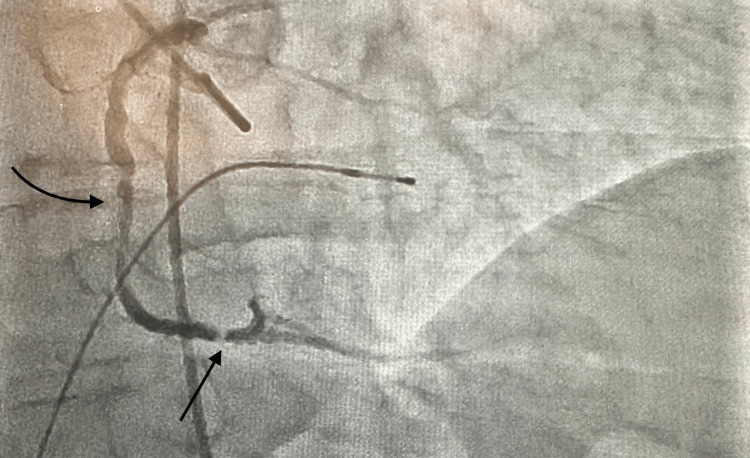
Coronary angiography revealing 2 lesions, the first in the proximal right coronary artery and another in the distal right coronary artery The curved arrow denotes a region of stenosis in the proximal right coronary artery. The lesion is diffusely calcified with 90% stenosis of the vessel. The straight arrow denotes a region of stenosis in the distal right coronary artery. The lesion is eccentric and calcified with 95% stenosis of the vessel.

Given the patient’s significantly elevated pro-BNP level and NYHA class III symptoms including unstable angina, cardiac catheterization was conducted. Catheterization revealed a heavily calcified obtuse marginal artery (OM1) lesion (Figure 6) as well as an apparent right coronary artery with severe disease (Figure 7). Interrogation of the right coronary artery led to the development of third-degree heart block. As a result, the vessel could not be treated with percutaneous coronary intervention (PCI), and the patient was subsequently referred for coronary artery bypass grafting (CABG).

Prior to beginning CABG, transesophageal echocardiography (TEE) was performed, revealing only mild mitral regurgitation, but also notable left ventricular hypertrophy (LVH), diastolic dysfunction, and mild global hypokinesis with an ejection fraction (EF) of about 45%.

Two-vessel CABG was performed using the greater saphenous vein and the left internal mammary artery (LIMA) as the bypass conduits. The greater saphenous vein quality was very good and was harvested endoscopically from the right leg. Examination of the heart revealed severe hypertrophy. Both the left and right ventricles were abnormally hypertrophied and notably, even both atria appeared to be thickened. Two anastomoses were performed: reverse saphenous vein graft to the right posterior descending artery (PDA) and LIMA to the OM1. Given the patient’s history of arrhythmia and high CHA2DS2-VASc score (CHF, Hypertension, Age ≥ 75, Diabetes, stroke, Vascular Disease, Age 65 to 74, and Sex Category score) the cardiothoracic surgeon performing the procedure elected to ligate the left atrial appendage and amputate the tip for pathologic evaluation for infiltrative cardiomyopathy. A pacemaker was implanted and two ventricular wires and an atrial wire were placed for heart pacing. The procedure was completed without complication with cardiac function adequate with postoperative EF about 45%, consistent with preoperative findings.

**Figure 8:**
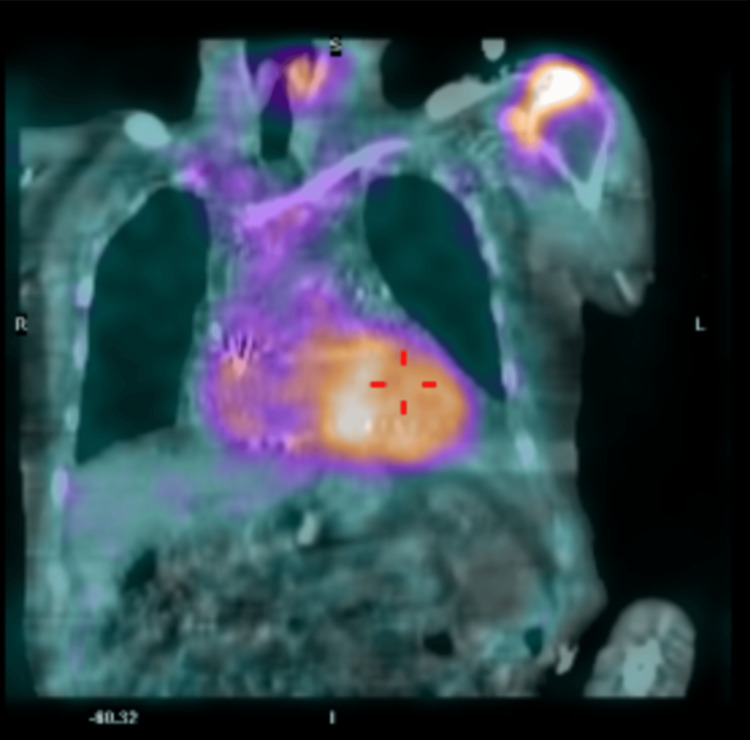
Coronal projection of fused SPECT/CT images displaying intense Tc99PYP tracer uptake in the interventricular septum SPECT/CT images acquired 60 minutes after the injection of radiotracer. SPECT: Single-photon emission computed tomography; Tc99m PYP: technetium pyrophosphate

**Figure 9:**
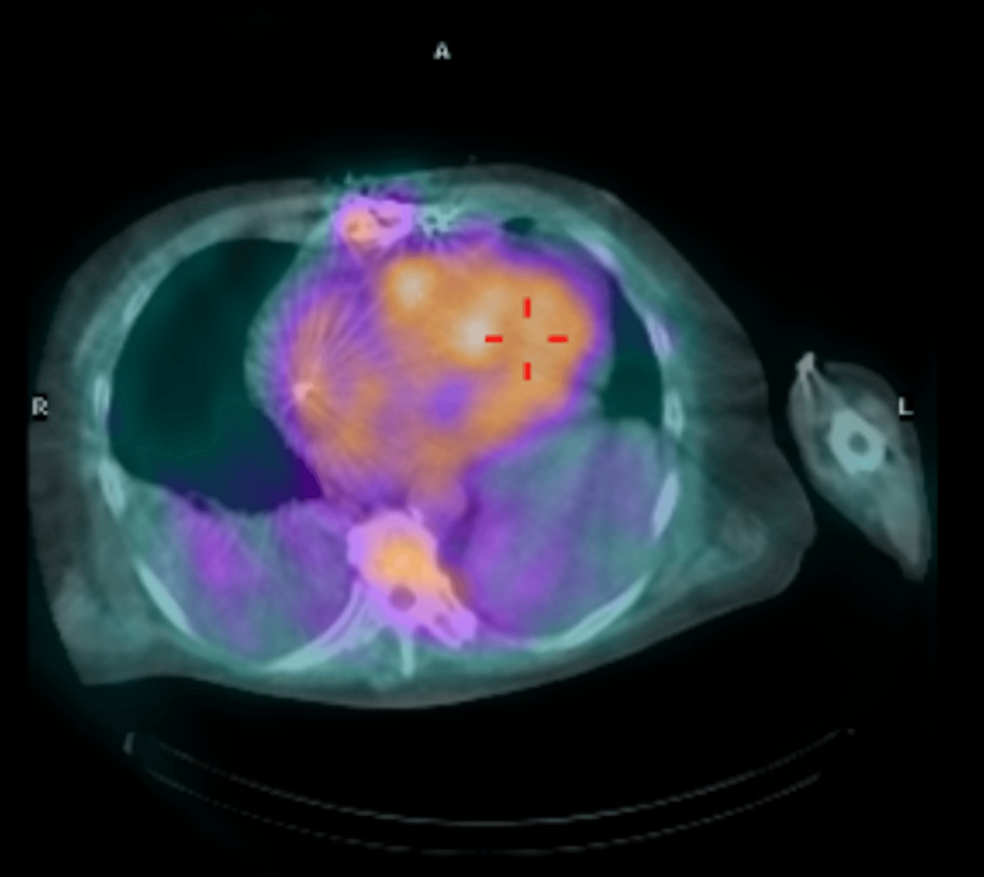
Axial projection of fused SPECT/CT images displaying intense Tc99PYP tracer uptake in the interventricular septum, mild uptake in the left ventricle lateral wall, and mild uptake in the right ventricular free wall SPECT/CT images acquired 60 minutes after the injection of radiotracer. SPECT: Single-photon emission computed tomography; Tc99m PYP: technetium pyrophosphate

**Figure 10:**
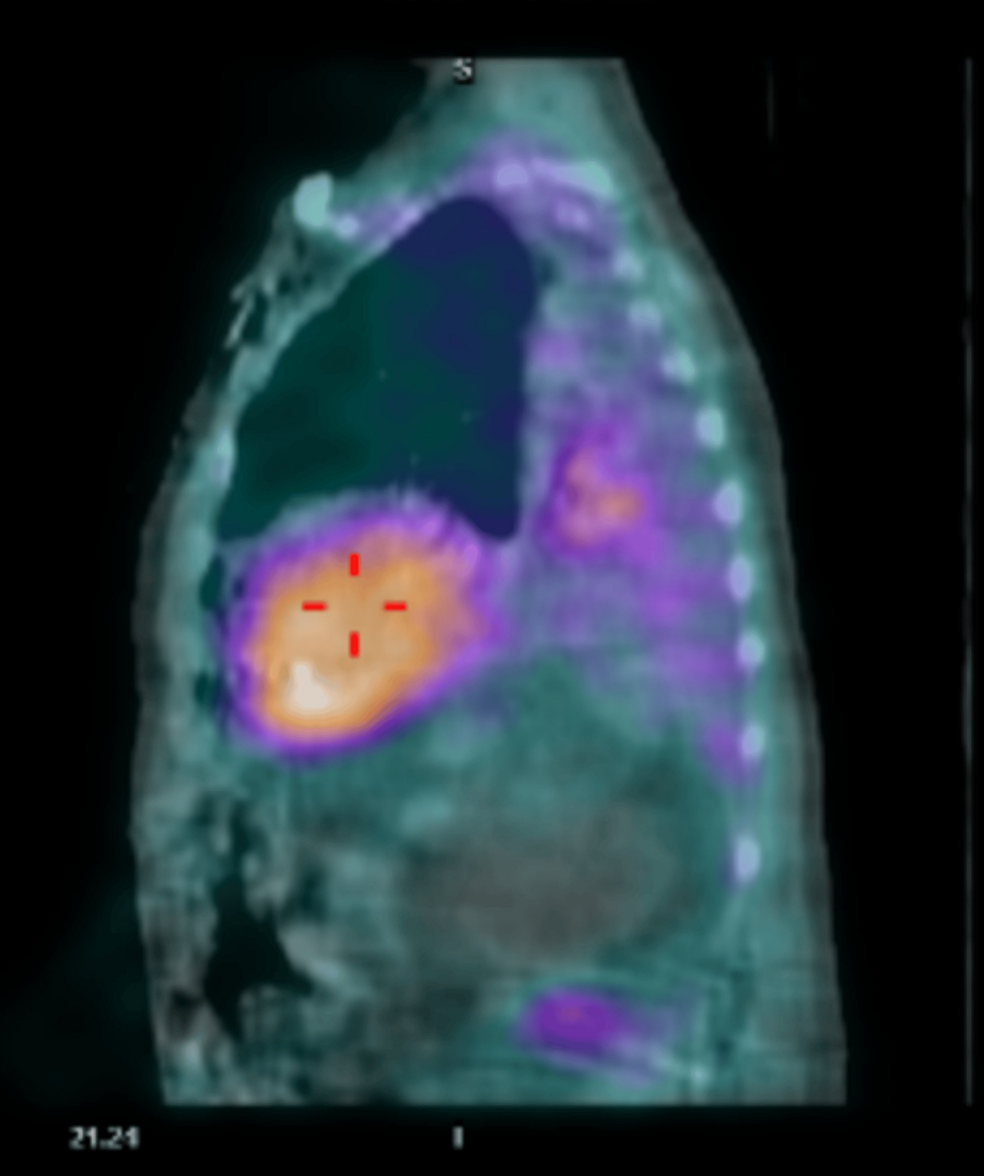
Sagittal projection of fused SPECT/CT images displaying mild Tc99m PYP uptake in the right ventricular free wall SPECT/CT images acquired 60 minutes after the injection of radiotracer. SPECT: Single-photon emission computed tomography; Tc99m PYP: technetium pyrophosphate

Pathologic evaluation of the cardiac tissue from the atrial appendage confirmed a diagnosis of ATTR. The cardiac tissue stained positive with Congo red indicating deposition of amyloid and was subsequently sent for further evaluation and subtyping. Additionally, the patient underwent myocardial imaging testing with technetium pyrophosphate (Tc99m PYP). Results of the testing further supported the diagnosis of ATTR-CA with abnormal 2+ uptake of radiotracer in the cardiac region equal to bone, HCL ratio (heart to contralateral lung ratio) of 1.76, and SPECT/CT (single-photon emission computed tomography) showing intense tracer uptake in the septum (Figure 8) and mild uptake in the left ventricle lateral wall (Figure 9) and right ventricular free wall (Figure 10). These results were consistent with diagnostic criteria of ATTR: tracer uptake of 2-3+, HCL ratio >1.5, and SPECT/CT showing diffuse tracer uptake in walls of the left and right ventricles. Subtyping of cardiac tissue was conducted using liquid chromatography-tandem mass spectrometry (LC-MS/MS) on peptides extracted from Congo red-positive, microdissected areas of the paraffin-embedded specimen. LC-MS/MS detected a peptide profile consistent with ATTR-type amyloid deposition. LC-MS/MS did not detect an amino acid sequence abnormality in the transthyretin protein, a finding most suggestive of age-related amyloidosis.

The patient’s postoperative hospital course proceeded without complication and the patient was evaluated by a heart failure specialist. The patient was prescribed 61 mg of Vyndamax (tafamidis) to be taken once a day and had prescriptions for Coreg (carvedilol) and lisinopril discontinued as they are not advantageous in ATTR. The patient was subsequently discharged home and since the hospitalization has had no recurrence of symptoms.

## Discussion

ATTR-CA is difficult to diagnose and may represent a greater proportion of cases of heart failure and cardiac arrhythmias than previously recognized [10]. Technetium-99m scintigraphy has been highlighted as a useful alternative to endomyocardial biopsy for diagnosis of ATTR cardiac amyloidosis with a sensitivity of 97% and specificity of 100% [11]. Despite advances in noninvasive cardiac imaging and growing recognition of the underdiagnosis of ATTR-CA, no cost-effective screening methods are routinely employed. ATTR-CA is often diagnosed late in the disease course and is associated with a significant impact on quality of life as well as a significant contributor to patient mortality. The median survival time after diagnosis of ATTRwt-CA is 3.80 years, stressing the need for early diagnosis and treatment [12]. Several disease-modifying drugs have been developed in recent years that are most effective when administered before significant symptoms of cardiac dysfunction manifest. In particular, the TTR stabilizer tafamidis has displayed promise as an effective treatment strategy. A multicentre placebo-controlled phase III study showed a reduction of all-cause mortality and hospitalizations in both ATTRm (mutant ATTR) and ATTRwt patients treated with oral tafamidis [13]. Validation of novel approaches to systems of care that operationalize screening of high-risk subpopulations is needed to facilitate earlier diagnosis and treatment. 

## Conclusions

Clinicians should maintain a high index of suspicion for ATTR amyloidosis in elderly individuals with treatment-resistant heart failure with preserved EF, even when presenting in acute settings. Percutaneous coronary intervention is generally preferred for the treatment of coronary artery disease in patients with concomitant cardiac amyloidosis. There are few published cases of successful bypass in patients with cardiac amyloidosis, prompting the need for greater research on outcomes to provide further evidence of its suitability for patients with unresolvable coronary artery disease via percutaneous coronary intervention.
